# NMR Based Cerebrum Metabonomic Analysis Reveals Simultaneous Interconnected Changes during Chick Embryo Incubation

**DOI:** 10.1371/journal.pone.0139948

**Published:** 2015-10-20

**Authors:** Yue Feng, Hang Zhu, Xu Zhang, Xuxia Wang, Fuqiang Xu, Huiru Tang, Chaohui Ye, Maili Liu

**Affiliations:** 1 State Key Laboratory of Magnetic Resonance and Atomic and Molecular Physics, Wuhan Center for Magnetic Resonance, Wuhan Institute of Physics and Mathematics, Chinese Academy of Sciences, Wuhan, 430071, P. R. China; 2 Department of Pharmaceutical Sciences, School of Pharmacy, University of Maryland, Baltimore, Maryland, 21201, United States of America; Mayo Clinic, UNITED STATES

## Abstract

To find out if content changes of the major functional cerebrum metabolites are interconnected and formed a network during the brain development, we obtained high-resolution magic-angle-spinning (HR-MAS) ^1^H NMR spectra of cerebrum tissues of chick embryo aged from incubation day 10 to 20, and postnatal day 1, and analyzed the data with principal component analysis (PCA). Within the examined time window, 26 biological important molecules were identified and 12 of them changed their relative concentration significantly in a time-dependent manner. These metabolites are generally belonged to three categories, neurotransmitters, nutrition sources, and neuronal or glial markers. The relative concentration changes of the metabolites were interconnected among/between the categories, and, more interestingly, associated with the number and size of Nissl-positive neurons. These results provided valuable biochemical and neurochemical information to understand the development of the embryonic brain.

## Introduction

Development of the central nervous system is tightly controlled by genetic factors, and modified, regulated and even limited by environmental factors. The molecular and cellular mechanisms for functions of individual genes, differentiation/growth or migration of brain cells, formation of neuronal networks, and effects of environment (e.g., nutritional conditions) have been extensively studied across the phyla with various techniques. All results point out that during normal development multidimensional dynamic networks must be precisely coordinated [1–4], in which the cerebral metabolites are certainly involved. Changes in cerebral metabolites have been used to interpret the biochemistry and physiology of the development of central nervous system (CNS) during the process of growth and maturation [5–10], such as cell differentiation, neuronal growth, specification and myelination. Therefore, a number of studies are focused on determination of neurochemical changes and metabolic profiles during brain development [5–19]. The major subjects include fetuses and child [5,8–11], rat embryos or pups [6,12–16], and chick embryos [7,17–19]. Analytical methods used in these studies include high performance liquid chromatography (HPLC), gas chromatography (GC), liquid nuclear magnetic resonance (NMR), and *in vivo* magnetic resonance spectroscopy (MRS). HPLC and liquid NMR are *in vitro* methods that need complex and labored sample preparation, such as tissue extracting, metabolite concentrating, etc. It is known that the sample preparation has a strong influence on reliability of the *in vitro* methods. The remarkable advantage of MRS is that the metabolites can be measured directly in a non-invasive manner. But only the higher concentrated compounds can be detected from the living system by MRS since the method has the low signal to noise ratio and low spectral resolution [9,10].

High resolution magic angle spinning (HR-MAS) NMR spectroscopy is a powerful *ex vivo* method for studying intact biologic tissue with minimum or even without any sample preparation [20]. In HR-MAS NMR, the sample is spun at several kHz along magic angle (54.7°) toward direction of the static magnetic field. This efficiently reduces the residual dipolar-interactions and the influence of magnetic susceptibility, leading to high-resolution NMR spectrum [21]. In contract to liquid NMR study of tissue extracts [6,13], HR-MAS NMR detects the tissue sample directly, therefore, information loss and artifacts introduced from extraction are largely avoided. Similar to solution NMR, HR-MAS NMR also suffers from spectral overlapping for complex biological system. However, this problem can be overcome because most multidimensional and spectral editing techniques used in NMR are applicable in HR-MAS NMR [22].

Principal component analysis (PCA), a multivariate data analysis method, is a powerful tool in classification of large and complex dataset, and thus has been widely used in metabonomics or metabolomics [23–25] for drug discovery and studies of pathophysiology, drug toxicity and adverse effects. PCA has been proved to be highly effective in classifying biological samples according to their NMR spectral profiles and thus the endogenous metabolite changes [24].

We had demonstrated that by using HR-MAS NMR it is possible to obtained the dynamic metabolic profile changes of three chick embryo brain regions, cerebrum, optic lobe and cerebellum, during the development, and successfully classified these three brain regions by their metabolites profile[17]. We now focused on a more important and relative larger brain region, cerebrum, and try to study that if the major metabolites content changes are interconnected with each other and formed a related network during chick embryo brain development. To prove this, 1D ^1^H HR-MAS NMR spectra of the cerebrum tissue of chick embryo were acquired from incubation day 10 to postnatal day 1, and the NMR dataset was analyzed using PCA. Within the examined time window, 12 biological important molecules were able to be identified and found the relative concentration significantly changed, thus formed a time-dependent metabonomic profile. More interestingly, the changes of relative concentration were happened in three categories of compounds, neurotransmitters, nutrition sources, and neuronal and glial markers, were highly interconnected among themselves and correlated with other developmental characters, including number and size of different brain cells at different stages. These results provided valuable biochemical and neurochemical information to understand the development of the embryonic brain.

## Experimental

### Animals

The thirty-five fertile eggs (Hy-Line Brown) were purchased from a local hatchery (Wuhan Chicken Hatchery, Wuhan, China). The incubation was conducted with temperature of 37°C and relative humidity of 60% in a Specific Pathogen Free (SPF, authorized by provincial government) animal laboratory of our Institute. Five eggs were randomly chosen at the incubation days of 10, 12, 14, 16, 18 and 20, and postnatal day 1 (approximated as day 22), respectively. These give rise to seven groups abbreviated as E10, E12, E14, E16, E18, E20 and P1, accordingly. The health statues of the embryos and the new born chicks were checked visually to exclude any sick objects. Also, the histological study and the similarity of their NMR spectra of the brain tissues showed all chick embryos are in good health conditions. Embryo brain samples were obtained according to the guidelines of national and provincial regulations. The surgery was performed under isoflurane anesthesia and finished within 60 sec, and all efforts were made to minimize suffering. Entire tissues of cerebrum were rapidly separated and frozen by liquid nitrogen, and then stored at -80°C freezer before NMR measurement.

### Ethics statement

Animal experimental procedures were performed according to the National Guidelines for Experimental Animal Welfare (Ministry of Science and Technology of People’s Republic of China, 2006) and approved by the Animal Welfare Committee of Wuhan Institute of Physics and Mathematics, Chinese Academy of Sciences, with permission from China Hubei Provincial Science and Technology Department.

### HR-MAS NMR spectroscopy

All NMR spectra were recorded on a Varian Unity INOVA 600 spectrometer (Palo Alto, CA) equipped with a Varian Nano-probe at 25°C. The cerebral tissue (about 10 mg, Figure A in S1 File) was dissected and packed into a 4 mm ceramic rotor with several drops of D_2_O to provide magnetic field lock for the NMR spectrometer. The samples were spun at 2000 Hz. One-dimensional (1D) ^1^H NMR spectra were acquired using a CPMG (Carr-Purcell-Meiboom-Gill) pulse sequence [26] with total spin echo time of 100 ms. The CPMG pulse sequence works as a transverse relaxation time (*T*_2_) filter to eliminate the broad peaks of macromolecules. Residual water signal was suppressed by using a soft pre-saturation pulse of 1.5s for all experiments. 128 transients were collected to ensure a sufficient signal-to-noise ratio (SNR). The acquisition data points were 16k with spectral width of 6000 Hz and recycle delay (RD) of 2s. A half-Gaussian window function (0.5 Hz) was applied to the time domain data, and zero-filed to 32k before Fourier transformation. For the purpose of metabolites identification or resonance assignment, two-dimensional (2D) ^1^H-^1^H correlation (COSY) experiment was carried out on two typical samples. It had a 2048 (F2) by 256 (F1) acquisition data set and 16 transients for each increment, and the spectral widths were 6000 Hz in both dimensions. Before Fourier transformation, a sine (0~π) window function was applied to both dimensions and time domain data point in F1 dimension was doubled by zero-filling.

### NMR data reduction and principal component analysis

All NMR data was processed on TopSpin 2.0 (Bruker Inc.). Each 1D CPMG NMR spectra, from chemical shift δ 4.7 to 0.7, was segmented into 100 integral regions (Δδ = 0.04 or 24 Hz). Resonances in the low filed region (δ > 4.7) were excluded because of very low SNR and less contribution to the results. Because the concentration of lactate (Lac) in brain tissue may be affected by glycolysis after death, and this process is very fast during the sample preparing, the regions belong to lactate (δ1.30−δ1.34, δ4.10−δ4.14) were not included. The resulting data matrix consists of 98 spectral regions by 35 samples. Before principal component analysis (PCA), each spectral data set was normalized to the total sum of the integrals, centered and scaled to Pareto Variance. PCA was performed on the normalized and scaled data matrix using the software SIMCA-P 11.0 (Umetrics, Sweden). The results were visualized by using the principal component scores (PCs) and loadings plots. Each point on the scores plot represents a trajectory of a reduced NMR spectrum and an individual sample. Each point on the loadings plot represents a single NMR spectral region. The score and loading plots are complementary: the former shows the differences between samples and the latter shows the spectral regions or chemical components being responsible for the differences [24].

### Cerebrum metabolites quantification and statistical analysis

For relative content measurement of brain metabolites, resonance of CH_3_ group of total creatine (Creatine and Phosphocreatine, tCr) at δ3.03 was selected as an internal reference [27,28]. After normalizing the total spectral area (100) in the region of δ0.7–δ4.7, the peak areas of tCr were 1.19±0.09 (n = 35). The linewidth at half-height is 1.39±0.23 Hz, corresponding to a *T*_2_ value of 236±43 ms as estimated from the linewidth and relaxation attenuation factor of 0.65±0.05 during the CPMG experiment with spin-echo time of 100 ms. The linewidths, *T*_2_ values and attenuation factors were 1.69±0.33 Hz, 196±39 ms and 0.59±0.06 for NAA at δ2.02, and 1.70±0.37 Hz, 196±46 ms and 0.59±0.07 for the center transition of Tau at δ3.43, respectively. These indicated that the normalized intensity of tCr was stable under current condition and could be used as internal reference, and variation of the relaxation attenuation factors (about 10%) were acceptable. Relative concentration of the target metabolite in the sample was derived using an equation

$$C_{\text{x}}=\;3A_{\text{x}}\;/\;N_{\text{x}}\cdot A_{\text{tCr}},$$

where *A*_x_ and *A*_tCr_ are NMR peak area of the metabolite (with *N*_x_ protons) and the total creatine (tCr, with 3 protons), respectively; a unit content of tCr (*C*_tCr_ = 1.0) is assumed. Statistical significance analysis was achieved using SPSS software package (SPSS, Chicago, USA).

### Histological assessments

Brain samples were taken from the storage and stored in the fixative solution (4% paraformaldehyde in phosphate-buffered solution (PBS) at pH 7.4.) store at 4˚C until being sectioned on a cryostat (Leica, Germany). Coronal brain sections were cut at a thickness of 10 μm and transferred onto glass slides coated with polylysine. For Nissl staining, the brain sections were transferred onto glass slides coated with polylysine. The slides were then incubated with 0.5% toluidine blue at room temperature for 15 min. Following rinses with tap water, the sections were dehydrated and mounted. The brain sections were examined under an Olympus BX51 microscope. The images cover an area of 327.7 × 435.8 μm^2^ with 400 amplification power. The numbers of Nissl-positive neuron were counted within the whole area of the images (N = 18~31) of 3~4 chick brains from each group. The size of the neuron was measured from 10 randomly chosen neurons within one image.

## Results

### HR-MAS NMR spectroscopy of cerebral tissue and assignment of metabolites

**Fig 1:**
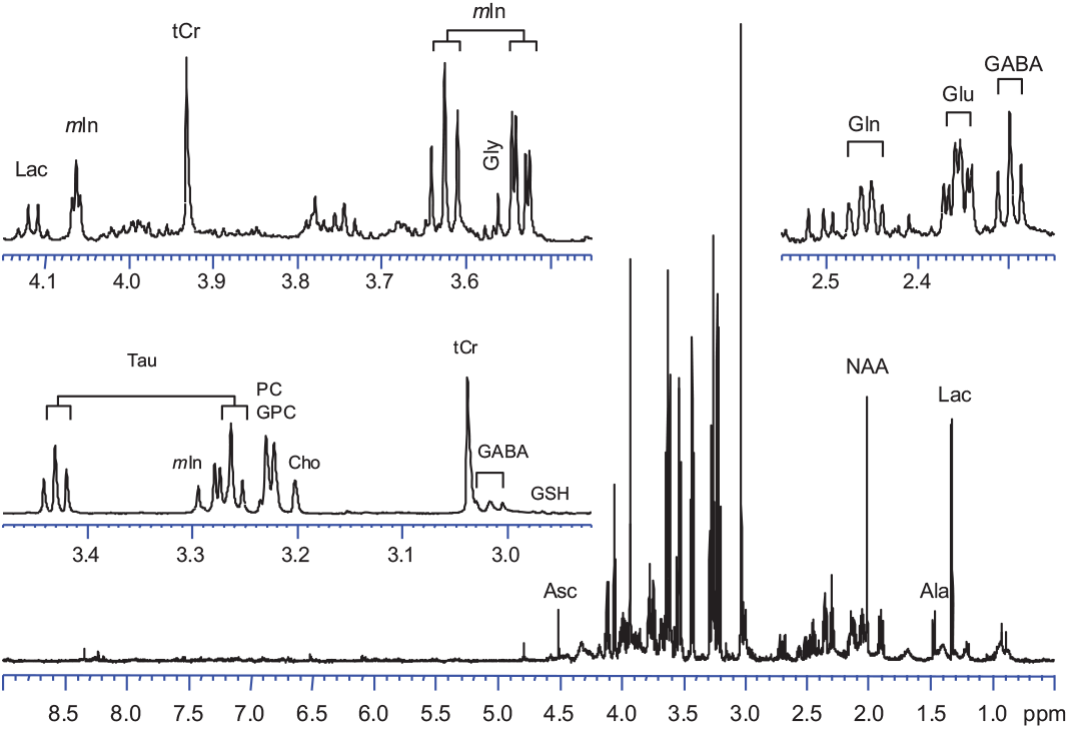
CPMG ^1^H NMR spectrum of the cerebral tissue of chick embryo in incubation day 20. Parts of the spectrum were enlarged and inserted. Major metabolites were identified and labeled based on their chemical shift, multiplicity and *J*-coupling connection of the signal. Abbreviations: Asc, Ascorbate; Ala, Alanine; NAA, N-Acetylaspartate; Cho, Choline; Lac, Lactate; GABA, γ-Aminobutyrate; Gln, Glutamine; Glu, Glutamate; Gly, Glycine; GSH, Glutathione; mI, myo-Inositol; Cr+PCr, total creatine; PC+GPC, Phosphoryl choline+Glycero phosphocholine; PEA, Phosphoryl ethanolamine; Tau, Taurine.

Fig 1 showed a typical HR-MAS CPMG spectrum of the cerebrum tissue of a chick embryo at incubation day 20 (E20). Vertical scales of three spectral regions, δ4.15−δ3.45, δ3.45−δ2.90 and δ2.55−δ2.35, were increased arbitrarily and plotted as inserts. The spectrum showed high efficiency in broad peak suppression, excellent baseline and resolution, which are comparable to a conventional solution NMR spectrum. This demonstrated that HR-MAS NMR is a powerful approach for studying metabolites in intact tissue without any sample pretreatment. Based on the *J*-coupling connection observed from the COSY spectrum (data not shown), data from literatures [16,27,29–32], we were able to identify 26 endogenous metabolites in the cerebral tissue (Table 1). The resonances of the major metabolites were labeled in Fig 1.

**Table 1:** Resonance Assignments with Chemical Shifts and Spin-Spin Coupling Patterns of Metabolites in Cerebral tissue of Chick Embryo. Chemical shifts are referenced to CH_3_ of Lactate at δ1.33.

No	Compound (abbreviation)	^1^H Chemical shift (multiplicity*)
**1**	Acetate (Ace)	1.92(s)
**2**	N-Acetylaspartate (NAA)	2.03(s); 2.50(m); 2.70(m); 4.40(m)
**3**	Alanine (Ala)	1.48(d); 3.78(q)
**4**	γ-Aminobutyrate (GABA)	1.90(m); 2.30(t); 2.99(t)
**5**	Ascorbate (ASC)	4.02(m); 4.52(d)
**6**	Asparate (Asp)	2.66(dd); 2.81(dd); 3.89(dd)
**7**	Choline (Cho)	3.20(s); 3.52(dd); 4.07(dd)
**8**	Total creatine (tCr)	3.03(s); 3.93(s)
**9**	Ethanolamine (EA)	3.15(m); 3.82(m)
**10**	Glutamate (Glu)	2.09(m); 2.36(m); 3.78(m)
**11**	Glutamine (Gln)	2.14(m); 2.46(m); 3.77(m)
**12**	Glutathione (Gsh)	2.94(dd); 2.98(dd); 4.58(dd)
**13**	Glycine (Gly)	3.56(s)
**15**	3-Hydroxybutyrate (3-HBA)	1.20(d); 2.31(dd); 2.41(dd); 4.16(q)
**16**	Isoleucine (Ile)	0.94(t); 1.01(d)
**17**	Lactate (Lac)	1.33(d); 4.12(q)
**18**	Leucine (Lec)	0.95(d); 0.96(d);
**19**	myo-Inositol (*m*In)	3.27(t); 3.53(dd); 3.63(t); 4.06(t)
**20**	Phosphoryl choline (tPC)	3.22(s)
**21**	Glyceroyl phosphocholine (tPC)	3.22(s)
**22**	Phosphoryl ethanolamine	3.22(m); 3.99(m)
**23**	Taurine (Tau)	3.25(t); 3.42(t)
**24**	Threonine (Thr)	1.33(d); 3.56(d); 4.26(q)
**25**	Tyrosine (Tyr)	6.90(d); 7.19(d)
**26**	Valine (Val)	0.98(d); 1.04(d)

* Multiplicity: s: singlet; d: doublet; dd: doublet of doublets; t: triplet; q: quartet; m: multiplet.

The brain tissue is known to degrade at room temperature. It is therefore necessary to find out a suitable time window, within which the tissue degradation is not significant. To test stability of the cerebral tissues, we carried out a time course NMR experiments, 30, 90 and 150 min after the sample taken out at room temperature. The brain tissue degradation was evidenced by intensity variations of certain small molecules after 90 min and become severe at 150 min (Figure B in S1 File). It was noticed that the amounts of leucine (Leu), isoleucine (Ile), valine (Val), alanine (Ala), γ-aminobutyrate (GABA), lactate (Lac), choline (Cho), glutamate (Glu) and glutamine (Gln) were increased, while that of N-acetylaspartate (NAA), phosphorylcholine (PC) and glycerol phosphorylcholine (GPC) were decreased. However, 3-hydroxybutyrate (3HBA), total creatine (tCr), taurine (Tau), myo-inositol (*m*In) and ascorbate (Asc) were less affected by the degradation. To minimize influence of tissue sample degradation, we kept HR-MAS NMR experiment to be finished within 30 min for all samples.

### Cerebral metabolic profiles during chicken embryo development

**Fig 2:**
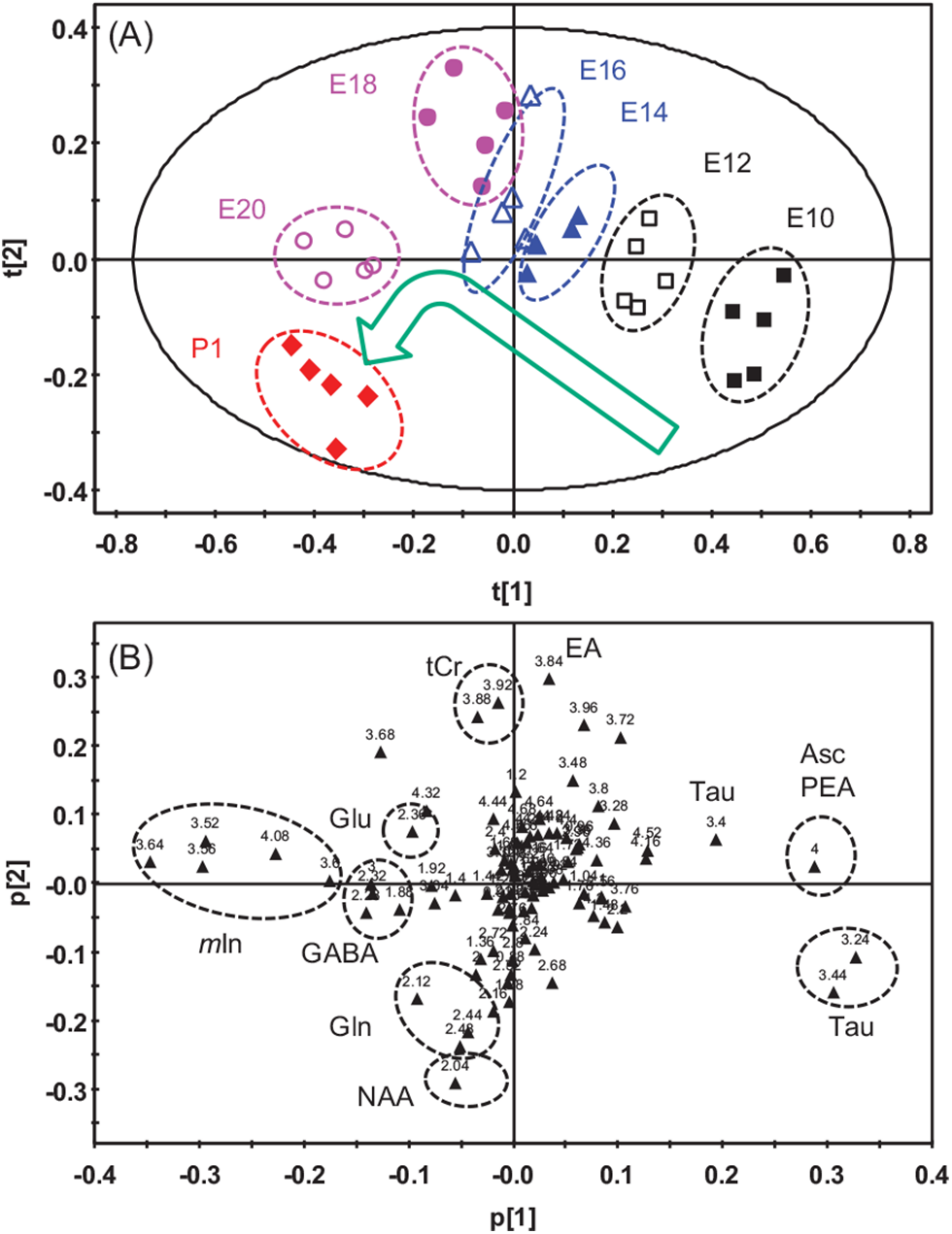
Scores plot (A) and loading plot (B) of the first two principal components of cerebrum obtained using SIMCA-P. The results were obtained using PCA based on the CPMG ^1^H NMR spectra of the cerebrum. The groups corresponding to incubation time and the metabolites that contribute to these classifications are labeled.

PCA was carried out on the normalized and scaled NMR data. Fig 2A showed PCA scores plot of the first two components (PC1 vs PC2), which explained 58.4% of the total variance and represented the cerebral metabolic profile variation during the incubation time. In the PCA scores plot, the metabolic profiles were generally separated into seven clusters, corresponding to the seven groups of the samples at different incubation time. The locations of the clusters changed steadily along a same direction from E10 to E18. We noticed that one of the data-point of the E16 group was a little bit away from the other four, which may be due to the individuality of embryo development. After E18, the profiles turned toward P1 and showed larger separation than the previous ones. Since each point in the PCA scores plot represented a trajectory of one NMR spectrum that consisted of resonances of NMR observable metabolites in the sample, variation in the PCA scores plot (Fig 2A) showed changes in the cerebral metabolic profile during the brain development of the chicken embryos.

The corresponding scatter loadings plot was showed in Fig 2B. The loading values (p[1] and p[2]) showed contribution of the spectral regions to the classification or clustering of the metabolic profiles during the brain development (Fig 2A). Referring to the loading plot, resonance assignment and the original NMR spectra, one can easily find out which chemicals contribute most to the classification or clustering. It can be seen from Fig 2B that positions of taurine resonances at δ3.24 and δ3.44 appeared at the right lower corner and matched the position of E10 group in the scores plot (Fig 2A). This implied that the E10 group had higher concentration of taurine than the other groups. Similarly, positions of *m*In (δ3.52, δ3.56, δ3.64 and δ4.06) and GABA (δ1.88, δ2.28, δ2.32 and δ3.00) (Fig 2B) indicated that these two chemicals were rich in the cerebrum at the later stage (E20) of the embryo and the first day of chick (P1).

### Relative content change of the cerebrum metabolites

**Fig 3:**
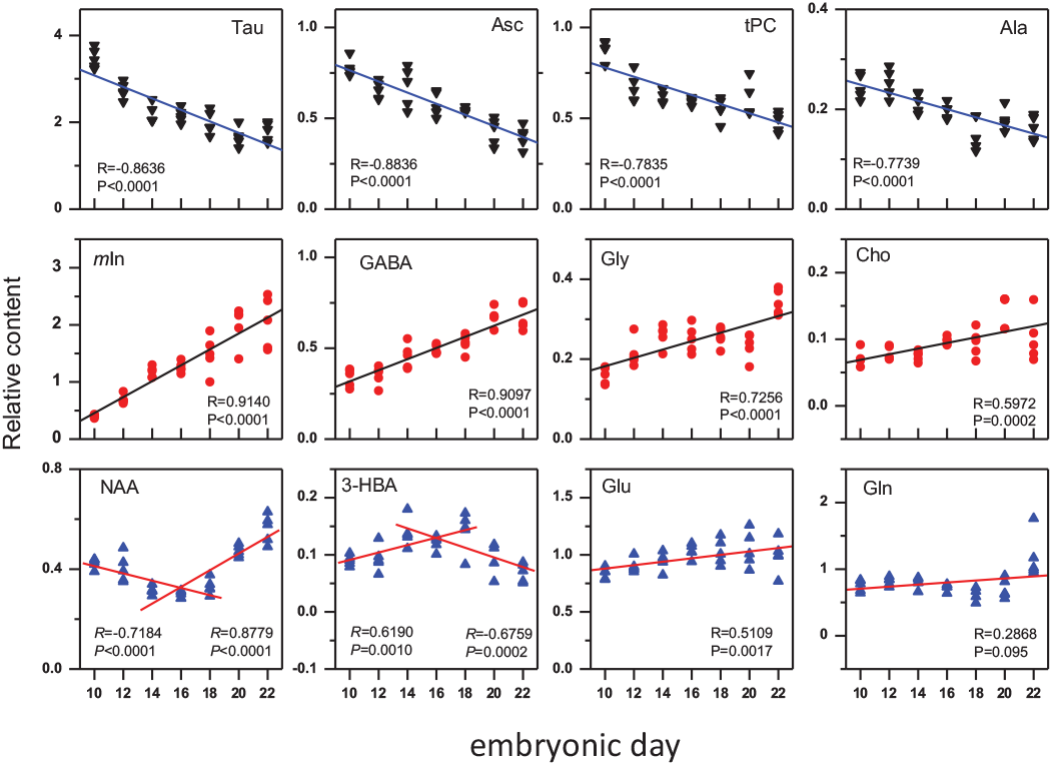
The relative concentration of major metabolites (mean + SD) in cerebral tissue changed with the development of chick embryo (embryonic day). The vertical scale is the relative concentration to total creatine. The star means that there was statistic significant differences between the group and the before (p < 0.05). The postnatal day 1(P1) is approximated as incubation day 22 (E22).

**Fig 4:**
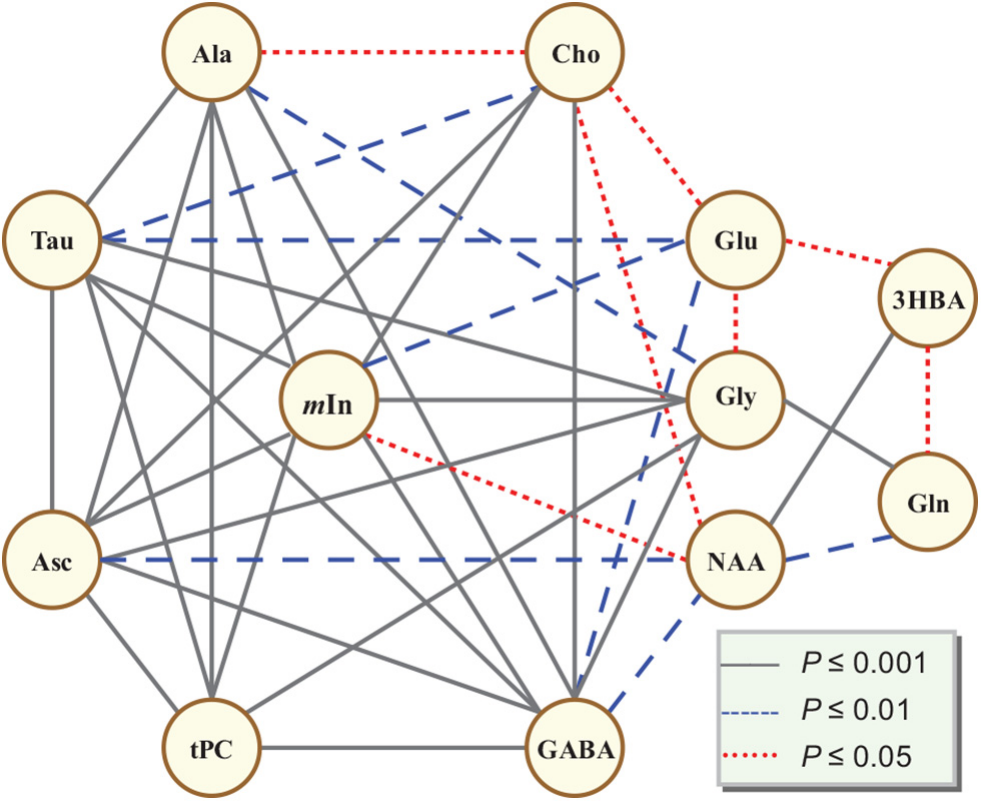
Diagram of the cerebral metabolites correlation network of chick embryo cerebrum during the development from incubation day 10 to 20 and postnatal day 1 (*P*≤ 0.05, N = 35×35).

The metabolite contents were measured relative to the total creatine content that had been widely used as internal reference in MRS [27,28]. Fig 3 showed relative concentration changes of twelve cerebral metabolites derive from the HR-NMR spectra during the period of study. The relative contents of *m*In, GABA, glycine (Gly), Cho and Glu were increasing, while Tau, Asc, tPC and Ala were decreasing. Gln, in general, showed less changes with exception of a significant increment at P1. Interestingly, NAA was decreasing from E10 to E16/E18 and increasing afterward. In contrast to NAA, 3HBA was increasing from E10 to E16/E18 and decreasing from E18 to P1. To find out if the metabolic changes are interconnected, we performed a linear fitting to the relative concentration of twelve metabolites. Figure C in S1 File demonstrated the typical correlations between ASC and *m*In/GABA, NAA and Gln/3HBA. It was noticed that the relative content changes of those metabolites were not only progressive (Fig 3), but also linear-correlated (Tables A and B in S1 File) and formed a complex network (Fig 4). We put *m*In in center of the network since it was significantly correlated to 9 metabolites with *P* < 0.0001 for Tau, Asc, tPC, Ala, GABA, Gly and Cho, and *P* < 0.05 for NAA and Glu. GABA was also correlated with 9 metabolites. Tau and Asc were correlated with 8 metabolites, Cho, Gly and Ala with 7, tPC, NAA and Glu with 6, Gln and 3HBA with 3.

### Changes of the neuron cell size and density

**Fig 5:**
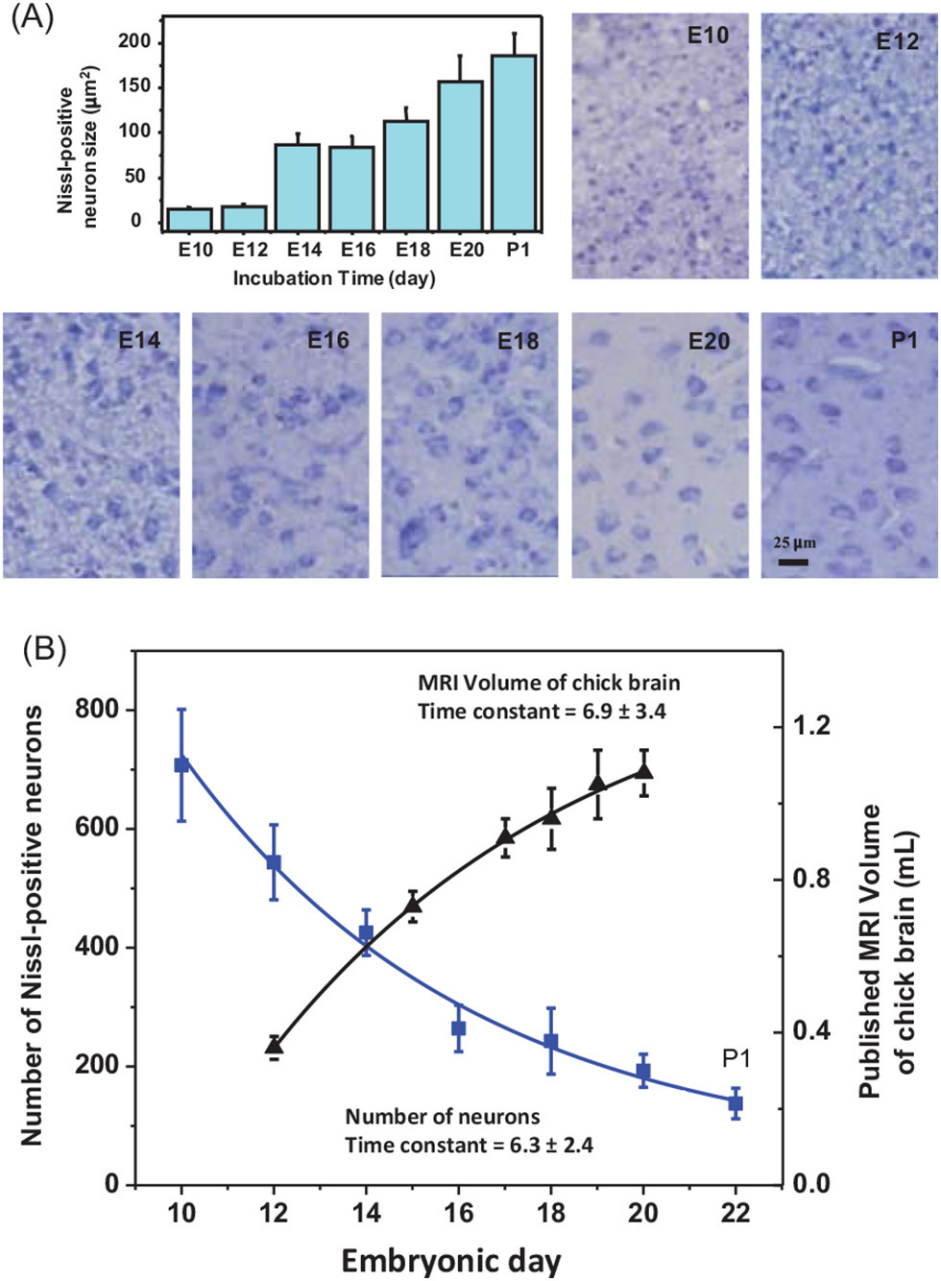
Histological microscopic images (×400) of the chick embryo brain, size and number of of Nissl-positive neurons at different embryonic day. (A) The histological microscopic images (×400) of the chick embryo brain and size (mean + SD, N = 10) of the Nissl-positive neurons at different embryonic day. (B) Plots of the number of Nissl-positive neurons (■) within the imaging area (327.7×435.8 μm^2^) and the chick brain MRI volume (▲) from reference [33] as function of the incubation time. The line symbols represented the exponential fittings.

Fig 5A showed the histological images and the sizes of Nissl-positive neurons (Table C in S1 File) as function of embryonic times, respectively. Numbers of the neuron (Table D in S1 File) counted from the whole imaging area (327.7 × 435.8 μm^2^) was plotted in Fig 5B, together with published chick brain volumes[33] measured using MRI. Fitting the data to an exponential equation (y = a+b×e^−t/T^), one get the time constant (*T*) of 6.3±2.4 from the neuron numbers and 6.9±3.4 for the brain volumes. The correlation between the neuron numbers and the relative contents of the metabolites were given in Figure D in S1 File.

## Discussion

### Relative content change of the cellular markers

The cerebral metabolites observed and quantified (Fig 3, Table 1, Table B in S1 File) are generally belonged to three categories, cellular markers, neurotransmitters and nutrition sources. Brain *m*In is distributed primarily in the glia cell within the central nervous system (CNS) and has been used as a marker of glia in MRI research [34]. Our results indicate that *m*In experiences about 5 times increment in relative concentration during the period of study and become one of the most abundant metabolites before/after hatching (Fig 3). As the glia cell marker, the fast increment of the *m*In may indicate dramatic proliferation of the glia cell from E10 to P1. This is in agreement with the result reported by Levi and Morisi [7]. During brain development, glia has a critical role in setting up the basic scaffolding of the brain. By interacting with specific cell adhesion molecules on the glial membrane, neurons migrate along appropriate glial processes and extend axons and dendrites using glia as “guide posts” to form proper synaptic connections [35,36]. This explains our observation that NAA, a well-known neuronal marker [5,8,11,37–43], experienced a slightly decease from E10 to E16, and fast increase from E16 to P1. The fast growth of glia cells may cause fast enlargement of the brain volume, which results in reduction of the neuron density. As a consequence, the relative content of NAA is slightly decreased from E10 to E16. When the “guide post” maturated and its number expanded, growth rate of the neuronal cell, as well as dendrites and synapses, is speedup. The histological experiments (Fig 5) showed the number and density of the Nissl-positive neurons was decreased from E10 to P1, while at the same time, the volume of the neurons was increased. Accordingly, the NAA content shows a postponed, relative to *m*In, but fast increment from E16 to P1. In a MRS study of human fetal and child brains, Kato et al. reported a similar phenomenon that NAA was decreased from 16 to 24 weeks’ gestation, and increased gradually from 24 weeks' gestation and remarkably from 40 weeks' gestation to 1 year of age [44]. The fast increment of NAA concentration, from incubation day 16 to postnatal day 1, may imply that the dendritic arborization, synaptogenesis and the neuron begin to have some function at the later stage of chicken brain development [38,44]. It has been reported that choline groups are involved in myelination of neurons [42] and also used as a marker of cell membrane proliferation [45]. Our results indicate that increment in relative content of choline is significantly correlated with that of the cell markers (NAA with *P* = 0.035 and *m*In with *P*<0.0001), and 5 other neuro-chemicals (GABA and Asc with *P*<0.001, Tau with *P*<0.01, Glu and Ala with *P*<0.05).

### Relative content change of neurotransmitters and other metabolites

Our results showed that the relative concentrations of the important neurotransmitters (GABA, Gly, Glu and Gln) in the cerebrum of chick embryo increase, but that of Tau decreases, with time and are maintained at high levels. As the brain develops, the neurons will gradually maturate, leading to the increase of the neurotransmitters, such as GABA, Gly, Glu and Gln. Among those, the relative contents of GABA, Gly and Glu are intercorrelated. Neuronal activity is frequently needed to establish proper neuronal contacts and for neuronal survive in the early developmental stages. Therefore, neurotransmitters also act as neurotrophic factors. For example, GABA treated embryonic chick cortical neurons in culture showed that GABA could promoted the proliferation and the differentiation of the neurons by affecting the length and branching of the neuritis as well as synaptogenesis [46]. It is therefore not surprising that the relative content increase of GABA is correlated with those of 9 other biochemicals, especially with *m*In (*P*<0.0001, a glia marker) and NAA (*P*<0.01, a neuronal marker), during the embryo brain development.

Taurine is an abundant and widely distributed amino acid, and is one of the important nutritional and functional ingredients. It is involved in diverse functions including modulation of neuronal excitability and calcium fluxes, membrane stabilization, differentiation of neural stem cells, maintenance of photoreceptor cells, antioxidation, osmoregulation, cell proliferation and immune system [15,47–49]. It was reported that taurine reduces the neuronal excitability and depresses the synaptic transmission in the inferior colliculus by activating glycine-gated chloride channels [50] and taurine acting on glycine receptors would serve to promote cortical circuit formation [51]. In addition, an antagonist blocking analysis demonstrated a developmental shift in the receptor target of taurine, from glycine receptors to GABA receptors [51]. There was evidence that physiological effects of taurine changed from excitatory to inhibitory due to variations in the intracellular Cl-concentration during development [51]. A similar phenomenon had been reported for glycine and GABA [52,53], both GABA and glycine provide excitatory action during early development and become inhibitory after neurons mature. This switch from excitation to inhibition is thought to result from a shift of intracellular chloride concentration from high to low [53]. Considering the genetically programmed and bidirectional functions of Tau, Gly and GABA, their contents are expected to increase during brain development of the chick embryo. Our data indicates that GABA and Gly are correlated (*P*<0.001) in the relative content increments, but their changes are inversely correlated with that of Tau. Because of high concentration (10.1~13.6 mg/100g) in egg yolk [49], taurine experienced a constant/linear decreasing in relative content from E10 to P1 (*P*<0.0001, *R* = −0.869), instead of increasing, but still remains at high level at least one day before/after hatching (Fig 3). This may imply that nutritional consumption of taurine is a dominating activity among all of its neurological roles during the brain development. Amount of the Tau consumption is likely proportional to the brain growth, or to the proliferation of the brain cells. This results in the significant correlation of the relative content changes between Tau and the other neuronal markers or transmitters, such as *m*In, Cho, Gly, GABA, Asc, tPC and Ala (all with *P*<0.0001), and Glu (*P*<0.01).

As an electron donor, ascorbate played an important role in CNS, such as antioxidant, free-radical scavenger and neuro-protection [54]. Ascorbate could be observed by in vivo MRS in human brain [55]. It was report that concentration of ascorbate in neuron is ten times higher than that in glia cell, thus, in the developing of rat brain, the content of ascorbate decreased along with the gliogenesis in the rat cortex [54]. In addition, ascorbate could promote the differentiation of CNS precursor cell into neurons and astrocytes, significantly increase synaptic activity of postmitotic neurons, and enhances the functional maturity of postmitotic neurons [56]. This was consistent with our result that the relative Asc content decrease is significantly correlated with that of the cellular markers (*m*In, NAA, Cho), transmitters (GABA, Gly), nutritional or energy ingredients (Tau Asc), and Ala as well (Fig 4).

### Correlation between the neuron numbers and metabolites

As the number of the Nissl-positive neurons were counted within a fixed area of 327.7×435.8 μm^2^, the results in Fig 5 demonstrated maturation and intensity changes of the neurons during the development. The mean neuron size was increased from 15.2±1.8 (E10) to 186.0±24.6 (P1) μm^2^, while the density decreased from (4.95±0.66) ×10^3^ (E10) to (0.96±0.18)×10^3^ (P1) neuron/μm^2^. The neuron density decrement is understandable since the chick brain volume is enlarged during incubation [33]. Fitting the numbers of the neuron counted from the whole imaging area and the published chick brain volumes measured using MRI [33] to an exponential equation (y = a + b×e^−t/T^), we get similar time constant (T) of 6.3±2.4 from the neuron numbers and 6.9±3.4 for the brain volumes (Fig 5B), respectively. This may imply that the majority of the neurons become mature unless apoptotic and newly generated neurons were balanced. Figure D in S1 File showed that the relationship between the neuron numbers and the relative contents of the metabolites during the incubation. The positive correlations were observed for Tau, ASC, tPC and Ala (*P* = 0.006~0.0052), and negative correlation for *m*In, GABA, Gly, Cho and Glu (*P* = 0.0008~0.033). Most of the metabolites in the negative correlation group are neurotransmitters, underwent significant increment in neuron. Apart from consumption of the initial abundant ones, such as Tau, very complex biochemical processes involved in the brain development, leading to dynamic changes for the chemicals. The correlations observed in this study confirms the hypothesis that the content changes of the major cerebrum metabolites of chick embryo are interconnected (Figs 3 and 5B; Figures C and D, and Table A in S1 File) and formed a network (Fig 4) during brain development.

## Conclusion

It is demonstrated that HR-MAS NMR can differentiate the cerebrum metabolic profile change of chick embryo at a two day interval from embryonic day 10 (E10) to 20 (E20), and postnatal day 1 (P1). Totally 26 metabolites were identified from the NMR spectra. It was found that relative content changes of 12 metabolites were not only correlated with the incubation time and Nissl-positive neuron numbers, but also interconnected and formed a complex network. These metabolites are generally belonged to three categories, neurotransmitters, nutrition sources, and neuronal or glial markers. The results may imply a simultaneous interconnected biochemical changes during chick embryo incubation, which provided valuable biochemical and neurochemical information to understand the development of the embryonic brain.

## Supporting Information

S1 File Additional Tables A-D and Figures A-D. **Table A.** Linear fitting parameters (R, P) between relative concentrations (N = 35×35) of the cerebral metabolites during embryo development from incubation day 16 to 20 and postnatal day 1. **Table B.** Relative metabolic levels and statistical analysis. **Table C.** Area of Nissl-positive neurons within the imaging area (327.7×435.8 μm2) during the embryo incubation. **Table D.** Number of Nissl-positive neurons (mean ± std, N = 18~31, same for all but only labeled in Tau) within the imaging area (327.7×435.8 μm2) during the embryo development from incubation. **Figure A.** Sketch of a chick embryo brain in incubation day 18 (dorsal view). All cerebrum tissue samples were dissected from the gray area in this study. **Figure B.** NMR spectra showed the metabolites of brain tissue degraded during the long experiment time. A, B and C were recorded at 30, 90 and 150 minutes after the tissue exposed at the experimental temperature, respectively. **Figure C.** Plots represent typical correlations of the relative concentration of *m*In and GABA as function of ASC, Gln and 2HBA as function of NAA. The linear fitting (line symbols) and the fitting parameters (*R*, *P*) are given in the plots. **Figure D.** Plots of relative concentration (mean ± std, N = 5) of 12 cerebral metabolites as function of the number of Nissl-positive neurons (mean ± std, N = 5, same for all but only labeled in Tau) within the imaging area (327.7×435.8 μm^2^) during the embryo development from incubation day 16 to 20 and postnatal day 1 (labeled in *m*In). The linear fitting (line symbols) and the fitting parameters (*R*, *P*) are given in the plots.
(PDF)
