# Supplementary material for: NMR Based Cerebrum Metabonomic Analysis Reveals Simultaneous Interconnected Changes during Chick Embryo Incubation
Source: PLoS One. 2015 Oct 20;10(10):e0139948. doi: 10.1371/journal.pone.0139948 (PMC4618859; doi:10.1371/journal.pone.0139948)
Supplement: S1 File — Table A. Linear fitting parameters (R, P) between relative concentrations (N = 35×35) of the cerebral metabolites during embryo development from incubation day 16 to 20 and postnatal day 1. Table B. Relative metabolic levels and statistical analysis. Table C. Area of Nissl-positive neurons within the imaging area (327.7×435.8 μm2) during the embryo incubation. Table D. Number of Nissl-positive neurons (mean ± std, N = 18~31, same for all but only labeled in Tau) within the imaging area (327.7×435.8 μm2) during the embryo development from incubation. Figure A. Sketch of a chick embryo brain in incubation day 18 (dorsal view). All cerebrum tissue samples were dissected from the gray area in this study. Figure B. NMR spectra showed the metabolites of brain tissue degraded during the long experiment time. A, B and C were recorded at 30, 90 and 150 minutes after the tissue exposed at the experimental temperature, respectively. Figure C. Plots represent typical correlations of the relative concentration of mIn and GABA as function of ASC, Gln and 2HBA as function of NAA. The linear fitting (line symbols) and the fitting parameters (R, P) are given in the plots. Figure D. Plots of relative concentration (mean ± std, N = 5) of 12 cerebral metabolites as function of the number of Nissl-positive neurons (mean ± std, N = 5, same for all but only labeled in Tau) within the imaging area (327.7×435.8 μm2) during the embryo development from incubation day 16 to 20 and postnatal day 1 (labeled in mIn). The linear fitting (line symbols) and the fitting parameters (R, P) are given in the plots. (PDF) [file pone.0139948.s001.pdf]

NMR Based Cerebrum Metabonomic Analysis Reveals Simultaneous Interconnected Changes during Chick Embryo Development

Yue Feng[1,2], Hang Zhu[1], Xu Zhang[1], Xuxia Wang[1], Fuqiang Xu[1], Huiru Tang[1], Chaohui Ye[1] and Maili Liu[1*]

1. State Key Laboratory of Magnetic Resonance and Atomic and Molecular Physics, Wuhan Center for Magnetic Resonance, Wuhan Institute of Physics and Mathematics, Chinese Academy of Sciences, Wuhan 430071, P. R. China.

2. Current address, Department of Pharmaceutical Sciences, School of Pharmacy, University of Maryland, Baltimore, Maryland 21201, United States

* E-mail: ml.liu@wipm.ac.cn

Table A. Linear fitting parameters (*R*, *P*) between relative concentrations (N=35×35) of the cerebral metabolites during embryo development from incubation day 16 to 20 and postnatal day 1

R / *P*	Tau	Asc	tPC	Ala	*m*In	GABA	Gly	Cho	NAA	3HBA	Glu	Gln
Tau		0.7973	0.8730	0.7475	-0.7973	-0.7626	-0.6039	-0.4705	-0.0906	-0.0161	-0.4601	-0.1522
Asc	<0.0001		0.7590	0.6950	-0.8258	-0.7939	-0.6074	-0.5613	-0.4358	0.2760	-0.2968	-0.2262
tPC	<0.0001	<0.0001		0.6964	-0.6587	-0.6344	-0.6420	-0.2585	-0.0460	0.0311	-0.3077	-0.2084
Ala	<0.0001	<0.0001	<0.0001		-0.6545	-0.6405	-0.5267	-0.3334	-0.1064	-0.0534	-0.2171	-0.0823
*m*In	<0.0001	<0.0001	<0.0001	<0.0001		0.8950	0.6949	0.7154	0.3950	-0.1667	0.4645	0.1688
GABA	<0.0001	<0.0001	<0.0001	<0.0001	<0.0001		0.5926	0.6588	0.4796	-0.2078	0.4947	0.2084
Gly	0.0001	0.0001	<0.0001	0.0012	<0.0001	0.0002		0.3121	0.3095	-0.0347	0.3644	0.5320
Cho	0.0043	0.0005	0.1338	0.0504	<0.0001	<0.0001	0.0680		0.3582	-0.1817	0.3965	-0.0992
NAA	0.6047	0.0089	0.7932	0.5428	0.0189	0.0036	0.0704	0.0346		-0.5987	0.0360	0.4684
3HBA	0.9267	0.1085	0.8593	0.7605	0.3385	0.2311	0.8432	0.2962	0.0001		0.3644	-0.3714
Glu	0.0054	0.0834	0.0722	0.2103	0.0049	0.0025	0.0314	0.0184	0.8371	0.0314		0.1686
Gln	0.3827	0.1914	0.2296	0.6385	0.3324	0.2295	0.0010	0.5707	0.0045	0.0280	0.3331	

Table B: Relative metabolic levels and statistical analysis

Times	Tau	Asc	tPC	Ala	*m*In	GABA	Gly	Cho	NAA	3-HBA	Glu	Gln	tCr
E10a	3.773	0.762	0.887	0.2375	0.418	0.378	0.136	0.06845	0.438	0.308	0.902	0.679	1.000
E10b	3.294	0.756	0.791	0.2736	0.406	0.360	0.139	0.07064	0.390	0.269	0.852	0.641	1.000
E10c	3.435	0.859	0.916	0.2169	0.398	0.276	0.177	0.05833	0.430	0.237	0.791	0.836	1.000
E10d	3.636	0.736	0.922	0.2678	0.364	0.298	0.162	0.09165	0.439	0.283	0.855	0.826	1.000
E10e	3.235	0.776	0.884	0.2298	0.434	0.385	0.181	0.06845	0.419	0.256	0.785	0.778	1.000
Average	3.475	0.778	0.880	0.245	0.404	0.339	0.159	0.072	0.423	0.270	0.837	0.752	1.000
Std	0.227	0.047	0.053	0.025	0.026	0.050	0.021	0.012	0.020	0.027	0.049	0.088	0.000
E12a	2.970	0.610	0.784	0.2166	0.833	0.369	0.275	0.08882	0.485	0.386	0.884	0.847	1.000
E12b	2.699	0.656	0.601	0.2531	0.627	0.266	0.211	0.06986	0.393	0.290	0.872	0.778	1.000
E12c	2.672	0.689	0.703	0.2718	0.675	0.379	0.198	0.07124	0.427	0.270	1.007	0.890	1.000
E12d	2.847	0.714	0.654	0.2866	0.662	0.336	0.205	0.07263	0.350	0.261	0.898	0.840	1.000
E12e	2.472	0.605	0.598	0.2347	0.684	0.402	0.184	0.09064	0.357	0.198	0.852	0.732	1.000
Average	2.732	0.655	0.668	0.253	0.696	0.350	0.215	0.079	0.403	0.281	0.903	0.817	1.000
Std	0.189	0.048	0.078	0.028	0.079	0.053	0.035	0.010	0.056	0.068	0.061	0.062	0.000
E14a	2.043	0.703	0.629	0.1981	1.298	0.552	0.213	0.06410	0.316	0.393	0.937	0.853	1.000
E14b	2.291	0.791	0.662	0.2320	1.084	0.480	0.269	0.08379	0.312	0.405	1.036	0.805	1.000
E14c	2.524	0.756	0.664	0.2340	1.079	0.461	0.286	0.07893	0.340	0.541	0.976	0.796	1.000
E14d	2.044	0.582	0.583	0.2173	1.191	0.388	0.255	0.07726	0.312	0.411	0.957	0.872	1.000
E14e	2.037	0.536	0.590	0.1903	1.219	0.396	0.272	0.07037	0.293	0.334	0.823	0.660	1.000
Average	2.188	0.674	0.625	0.214	1.174	0.456	0.259	0.075	0.314	0.417	0.946	0.797	1.000
Std	0.217	0.110	0.038	0.020	0.094	0.067	0.028	0.008	0.017	0.076	0.078	0.083	0.000
E16a	2.130	0.642	0.567	0.1832	1.213	0.526	0.212	0.09257	0.314	0.354	1.102	0.762	1.000
E16b	2.380	0.536	0.596	0.2004	1.236	0.471	0.297	0.10582	0.301	0.398	1.026	0.637	1.000
E16c	1.960	0.500	0.589	0.2012	1.395	0.474	0.225	0.09793	0.309	0.387	1.076	0.734	1.000
E16d	2.076	0.652	0.609	0.2176	1.291	0.514	0.268	0.09971	0.283	0.302	1.024	0.772	1.000
E16e	2.227	0.559	0.615	0.1802	1.145	0.479	0.250	0.09109	0.284	0.378	0.941	0.735	1.000
Average	2.154	0.578	0.595	0.197	1.256	0.493	0.250	0.097	0.298	0.364	1.034	0.728	1.000
Std	0.159	0.067	0.019	0.015	0.094	0.026	0.034	0.006	0.014	0.038	0.062	0.054	0.000
E18a	2.326	0.564	0.595	0.1870	1.417	0.558	0.280	0.10222	0.377	0.431	1.173	0.668	1.000
E18b	2.205	0.564	0.613	0.1866	1.490	0.581	0.250	0.09766	0.339	0.480	1.104	0.588	1.000
E18c	1.673	0.542	0.546	0.1269	1.644	0.451	0.255	0.12123	0.321	0.443	0.901	0.588	1.000
E18d	1.878	0.544	0.455	0.1165	1.000	0.520	0.220	0.06716	0.322	0.519	1.007	0.491	1.000
E18e	1.886	0.531	0.547	0.1420	1.895	0.532	0.275	0.08180	0.292	0.250	0.949	0.724	1.000
Average	1.994	0.549	0.551	0.152	1.489	0.528	0.256	0.094	0.330	0.425	1.027	0.612	1.000
Std	0.266	0.015	0.061	0.033	0.329	0.049	0.024	0.021	0.031	0.104	0.111	0.089	0.000
E20a	1.568	0.336	0.530	0.1552	1.946	0.741	0.260	0.16007	0.470	0.158	0.866	0.812	1.000
E20b	2.005	0.480	0.746	0.2133	2.243	0.679	0.259	0.15923	0.489	0.353	1.261	0.901	1.000
E20c	1.684	0.506	0.644	0.1783	2.183	0.599	0.227	0.16019	0.457	0.336	1.149	0.623	1.000
E20d	1.409	0.456	0.534	0.1745	1.403	0.673	0.181	0.11547	0.503	0.259	1.009	0.632	1.000
E20d	1.647	0.370	0.536	0.1702	2.168	0.669	0.241	0.11603	0.446	0.259	0.956	0.560	1.000
Average	1.662	0.430	0.598	0.178	1.989	0.672	0.234	0.142	0.473	0.273	1.048	0.706	1.000
Std	0.219	0.073	0.096	0.021	0.346	0.050	0.032	0.024	0.023	0.078	0.157	0.144	0.000
P1a	2.008	0.473	0.520	0.1834	2.532	0.756	0.380	0.15912	0.629	0.163	1.031	0.948	1.000
P1b	1.961	0.373	0.436	0.1359	2.082	0.637	0.310	0.10908	0.518	0.154	0.769	1.019	1.000
P1c	1.526	0.411	0.416	0.1403	1.603	0.626	0.370	0.07840	0.490	0.261	1.183	1.759	1.000
P1d	1.601	0.422	0.539	0.1637	1.563	0.596	0.337	0.06948	0.578	0.245	0.989	1.169	1.000
P1e	1.833	0.315	0.498	0.1898	2.424	0.748	0.317	0.09142	0.594	0.216	0.994	0.987	1.000
Average	1.786	0.399	0.482	0.163	2.041	0.673	0.343	0.102	0.562	0.208	0.993	1.177	1.000
Std	0.215	0.059	0.053	0.024	0.450	0.074	0.031	0.035	0.057	0.048	0.148	0.336	0.000

Table C. Area of Nissl-positive neurons within the imaging area (327.7×435.8 μm^2) during the embryo incubation

Samples	E10	E12	E14	E16	E18	E20	P1
#1	17.6	20.5	78.1	71.2	105.9	141.5	177.1
#2	14.5	16.0	106.9	73.1	106.6	152.1	143.5
#3	17.4	22.7	75.0	89.4	131.8	188.2	168.0
#4	14.9	15.2	87.9	90.7	135.7	189.6	160.3
#5	14.2	11.5	78.8	79.6	121.1	142.8	218.4
#6	17.1	15.7	84.3	95.8	106.3	208.0	199.6
#7	16.4	22.9	100.5	84.0	111.1	144.9	213.0
#8	14.1	16.2	98.1	76.6	111.3	117.9	176.3
#9	13.4	16.4	88.0	63.4	115.5	150.9	204.0
#10	12.7	15.6	68.9	107.6	80.8	126.4	200.1
Average	15.2	17.3	86.6	83.1	112.6	156.2	186.0
Std	1.8	3.6	12.1	13.1	15.3	29.4	24.6

Table D. Number of Nissl-positive neurons (mean ± std, N=18~31, same for all but only labeled in Tau) within the imaging area (327.7×435.8 μm^2) during the embryo development from incubation

Samples	E10	E12	E14	E16	E18	E20	P1
#1	877	459	403	251	183	201	117
#2	697	563	433	296	187	176	111
#3	916	597	430	357	201	200	98
#4	652	544	419	304	217	168	136
#5	648	568	418	307	197	205	111
#6	630	567	385	274	192	198	128
#7	745	619	471	311	215	187	172
#8	821	571	414	310	194	121	141
#9	652	487	420	239	363	163	174
#10	675	437	370	203	270	208	175
#11	625	519	458	225	255	213	143
#12	695	472	451	204	256	200	105
#13	616	464	394	226	261	209	124
#14	743	411	436	215	302	177	138
#15	647	445	453	257	322	196	162
#16	781	540	394	298	282	193	108
#17	566	575	379	249	341	172	175
#18	746	553	371	259	270	205	152
#19		580	421	239	207	262	147
#20		576	363	242	171	204	
#21		603	427	201	185	173	
#22		522	385	261	269	160	
#23		624	417	288		169	
#24		465	397	296		211	
#25		579	468	240		247	
#26		590	490	270		198	
#27		595	504	247			
#28		638	482	311			
#29		610	472	279			
#30			396				
#31			468				
Average	707.3	543.9	425.5	264.1	242.7	192.9	137.7
Std	94.1	63.1	38.3	39.0	55.6	27.8	25.7

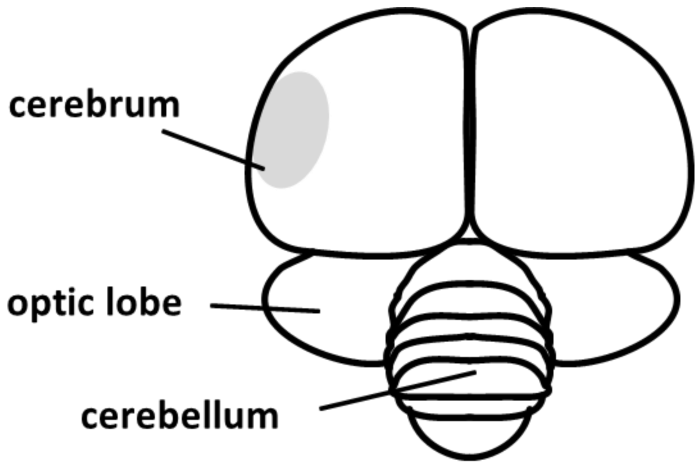

Figure A. Sketch of a chick embryo brain in incubation day 18 (dorsal view). All cerebrum tissue samples were dissected from the gray area in this study.

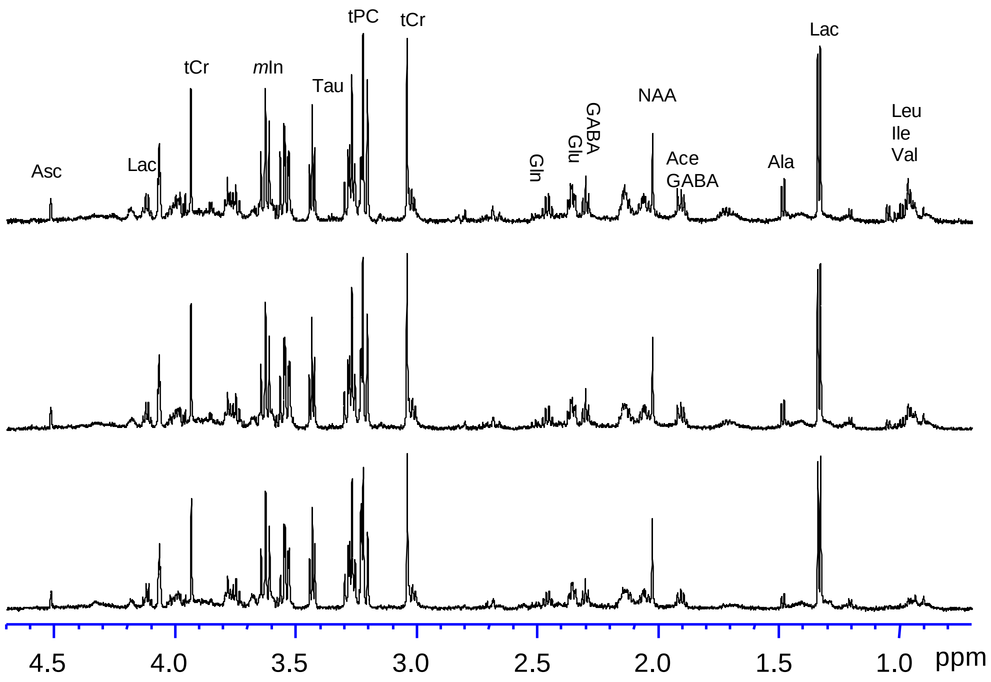

Figure B. NMR spectra showed the metabolites of brain tissue degraded during the long experiment time. A, B and C were recorded at 30, 90 and 150 minutes after the tissue exposed at the experimental temperature, respectively.

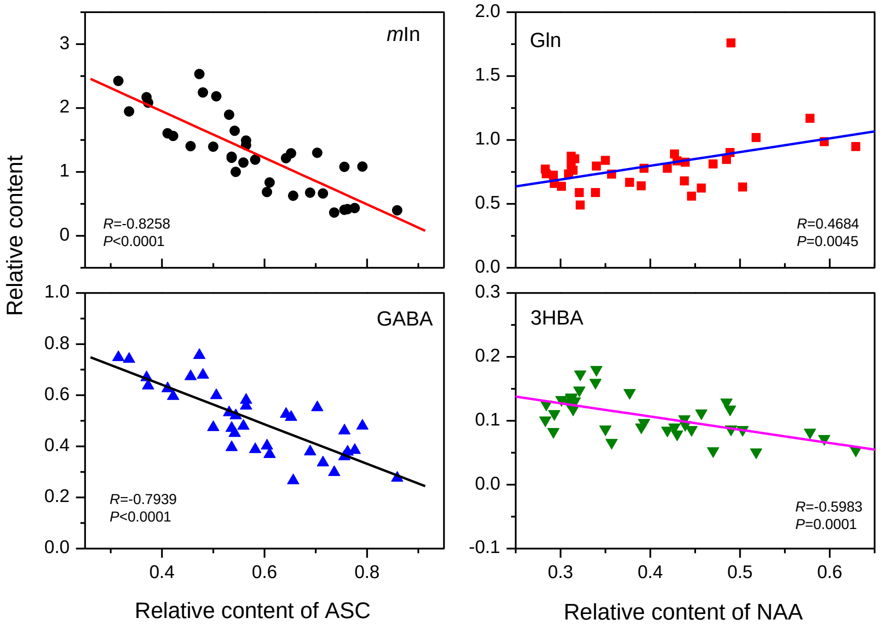

Figure C. Plots represent typical correlations of the relative concentration of *m*In and GABA as function of ASC, Gln and 2HBA as function of NAA. The linear fitting (line symbols) and the fitting parameters (*R*, *P*) are given in the plots.

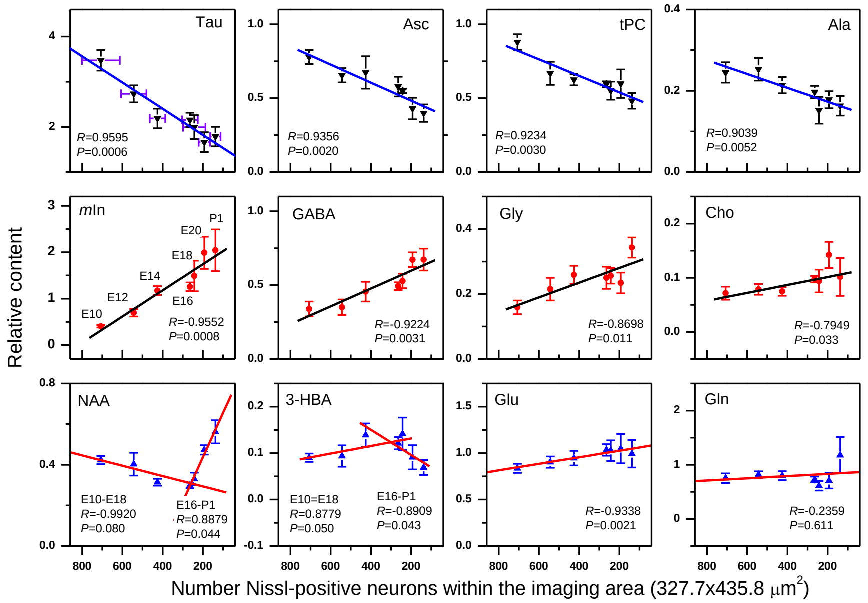

Figure D. Plots of relative concentration (mean ± std, N=5) of 12 cerebral metabolites as function of the number of Nissl-positive neurons (mean ± std, N=18~31, same for all but only labeled in Tau) within the imaging area (327.7×435.8 μm^2) during the embryo development from incubation day 16 to 20 and postnatal day 1 (labeled in *m*In). The linear fitting (line symbols) and the fitting parameters (*R*, *P*) are given in the plots.
